# Ocular Structural and Vascular Changes in Patients with Severe Asymptomatic Carotid Disease After Undergoing Carotid Endarterectomy (CEA) and Carotid Artery Stenting (CAS)

**DOI:** 10.3390/diagnostics15141826

**Published:** 2025-07-21

**Authors:** Foteini Xanthou, Anna Dastiridou, Athanasios Giannoukas, Miltiadis Matsagkas, Chara Tzavara, Athanasios Chaidoulis, Sofia Androudi, Evangelia E. Tsironi

**Affiliations:** 1 Ophthalmology Department, Faculty of Medicine, School of Health Sciences, University of Thessaly, University Hospital of Larissa, 41110 Larissa, Greece; anna.dastiridou@gmail.com (A.D.); androudi@otenet.gr (S.A.); e_tsironi@hotmail.com (E.E.T.); 2 Vascular Surgery Department, Faculty of Medicine, School of Health Sciences, University of Thessaly, University Hospital of Larissa, 41110 Larissa, Greece; 3 Biostatistics Department, National and Kapodistrian University of Athens, 15784 Athens, Greece

**Keywords:** carotid artery stenosis, carotid endarterectomy, carotid artery stenting, OCT, OCT-angiography, choriocapillaris vascular density, retinal vascular density, retinal embolization, choroidal thickness

## Abstract

**Background/Objectives**: This study aimed to prospectively assess the incidence of retinal embolization and to evaluate the vascular and structural changes in the retina and choroid in 52 patients with asymptomatic severe carotid artery disease who underwent carotid artery revascularization. **Methods**: In our study, 35 patients underwent carotid endarterectomy (CEA) and 17 underwent carotid artery stenting (CAS). Biomicroscopy, fundoscopy, optical coherence tomography (OCT), and OCT-angiography (OCTA) were performed at baseline and 1 month after revascularization. **Results**: The subfoveal choroidal thickness (SFCT), peripapillary choroidal thickness inferior to the optic nerve head (ppCTi), total overall average retinal vascular density (rVDtot), and total overall average choriocapillaris vascular density (ccVDtot) of the eyes ipsilateral to the operated carotid artery increased significantly after revascularization, whereas a statistically significant increase was also found in the SFCT, rVDtot, and ccVDtot of the contralateral eyes in the overall cohort. Comparing the two study groups, we found that the SFCT, superior and inferior peripapillary choroidal thicknesses (ppCTs, ppCTi), rVDtot, and ccVDtot increased in both groups after revascularization, but significantly only in the CEA group. Furthermore, the temporal choriocapillaris vascular density (ccVDt) increased significantly after revascularization in both groups to a similar degree. **Conclusions**: Carotid artery revascularization led to a statistically significant increase in retinal and choroidal vascular densities, which indicates significantly improved ocular perfusion. The analysis of the findings of the two study groups suggests the superiority of CEA in terms of improving ocular perfusion in asymptomatic severe carotid artery disease. The rate of retinal embolization was similar in both surgical groups.

## 1. Introduction

Carotid artery disease, as a manifestation of atherosclerosis, is a major risk factor for vascular events in multiple locations, including ipsilateral ischemic stroke and ocular ischemic syndrome, and therefore represents a major cause of morbidity and mortality. Carotid artery stenosis has become a prominent public health concern. The estimated rate of ipsilateral carotid-related acute ischemic stroke in asymptomatic patients with severe (70–99%) carotid artery stenosis is 4.7% over 5 years [1]. Overall, carotid artery stenosis is the cause of 10–20% of ischemic strokes, which are the second most common cause of death worldwide [2]. It is important to distinguish between asymptomatic and symptomatic carotid artery stenosis, as they are associated with different stroke risks [3]. A symptomatic carotid lesion is referred to as a symptomatic lesion after a stroke, transient ischemic attack, or amaurosis fugax. Moreover, ocular signs and symptoms may be the first manifestations of carotid artery disease [4,5], as the blood supply to the eye is mostly provided by branches of the ophthalmic artery, which is the first branch of the internal carotid artery (ICA). Notably, retinal embolization is associated with an increased risk of stroke [6,7].

Furthermore, asymptomatic carotid stenosis has been associated with neurocognitive decline, possibly as the result of silent microinfarctions, which have been found in 17–33% of patients with asymptomatic carotid disease [8]. However, the mechanisms contributing to the development of vascular cognitive impairment associated with carotid artery stenosis are multifaceted and not fully understood.

The objective of carotid artery revascularization is to prevent primary or secondary strokes. The methods available for carotid artery revascularization include carotid endarterectomy (CEA), intraluminal restoration with carotid artery stenting (CAS), and transcarotid artery revascularization (TCAR). Carotid endarterectomy (CEA) was the first method that was developed; it was first performed in 1954 and remained the only method for about 40 years. Currently, CEA is the standard treatment. CEA involves exposure of the carotid artery and mechanical removal of plaque, most typically from the carotid bulb and the proximal internal carotid artery, via a neck incision. CAS, which is an endovascular, minimally invasive procedure, involves femoral (less often radial) puncture, catheter introduction to the carotid artery proximal to the site of stenosis, neuroprotection system (distal or proximal) activation, balloon dilatation of the stenotic area, and finally, stent implantation for endovascular lumen reconstruction. If performed with the routine use of neuroprotection and second-generation stent (mesh-covered, antiembolic) implantation, CAS is safe and at least as effective as CEA in stroke prevention. The third method is the hybrid procedure of transcarotid artery revascularization (TCAR), which has emerged as a valid alternative to both open CEA and transfemoral/transradial CAS. In TCAR, a direct puncture approach involves accessing the carotid artery through a small incision in the neck. Subsequent procedural steps are similar to CAS. This technique enables a more direct and less invasive approach to revascularization, potentially reducing complications compared with traditional methods. These procedures effectively reduce the risk of first and recurrent ischemic stroke. Choosing between the aforementioned carotid revascularization procedures depends on several factors, including the patient’s anatomy, age, gender, and procedural risk.

It is currently not known whether CEA, CAS, or both lead to a change in ocular perfusion. This could be observed using OCT-angiography, a new noninvasive imaging modality that can provide qualitative and quantitative information about retinal and choroidal microcirculation [9]. Therefore, the objective of this study was to examine whether there are any differences in the ipsilateral choroidal and retinal perfusion or retinal embolization after CEA vs. CAS, and to investigate the effect of revascularization on the contralateral eye.

## 2. Materials and Methods

This single-center prospective study was conducted at the University Hospital of Larissa, Greece, from October 2018 to December 2022. Patients were eligible to participate in this study if they had asymptomatic, severe (>70–99%) stenosis of the internal carotid artery (ICA) and were scheduled for carotid artery revascularization. Asymptomatic was defined as the absence of symptoms (stroke, TIA, and amaurosis fugax) for at least 6 months. The percentage of the carotid stenosis was evaluated by both ultrasound and OCT-angiography. They were divided into two groups, one undergoing carotid endarterectomy (CEA) and the second undergoing carotid artery stenting (CAS). The vascular surgeons selected the surgical procedure. This study’s protocol adheres to the Declaration of Helsinki and was approved by the University Hospital of Larissa Review Board. Before enrolling, eligible participants were required to sign an informed consent form.

Patients who met any of the following criteria were excluded: symptomatic, severe carotid artery stenosis; uncontrolled arterial hypertension; poor glycemic control; best-corrected visual acuity (BCVA) < 0.7; ocular hypertension; glaucoma; severe non-proliferative or proliferative diabetic retinopathy; prior pan-retinal photocoagulation; history of retinal vein or artery occlusion; history of posterior uveitis; ocular ischemic syndrome; prior or current treatment with intravitreal anti-VEGF or steroid injections; refractive error with spherical equivalent > 3 diopters; corneal opacification; dense cataract; prior ocular surgeries other than uneventful cataract extraction.

The patients were examined 1–3 days before and 1 month after revascularization. Preoperative and postoperative ophthalmological examinations were performed at the same time of day (within a 3 h span) to avoid significant diurnal variations, and all patients had normal blood pressure at the time of examination. BCVA, tonometry, biomicroscopy, and fundoscopy were performed to exclude ocular pathology. Additionally, macular OCT, OCT of the optic nerve head, and OCT-angiography (OCTA) of the retina and choriocapillaris were included in each ophthalmological examination. OCT and OCTA are suitable for the quantitative estimation of indicators such as the choroidal thickness, retinal structure, and retinal and choroidal vascular densities, which are currently used as biomarkers for ischemic ophthalmopathy [10]. In addition, fundus color images were obtained to further document the absence of ocular diseases.

**Scheme 1:**
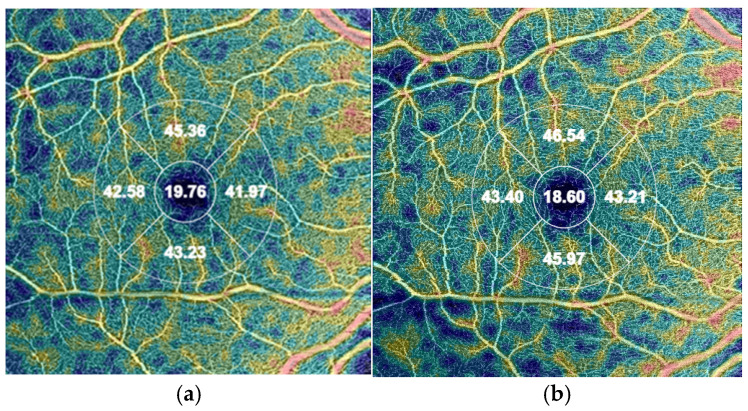
(**a**): pre-procedural vascular density map of superficial retinal vascular plexus; (**b**): post-procedural vascular density map of superficial retinal vascular plexus.

OCT and OCTA imaging were performed using the Triton Plus swept source OCT (Topcon Inc., Tokyo, Japan) with its 100 kHz scanning speed and wavelength of 1050 nm, and the coupled SMARTTrackTM real-time eye-tracking system. The scanning protocols included a 6 × 6 mm 3D OCT-angiography scan centered on the fovea with a resolution of 320 × 320 pixels and a 3D OCT 7 × 7 mm scan with a resolution of 512 × 256 pixels (Scheme 1a,b). OCT retinal macular thickness (MT) and choroidal thickness (CT) were quantified using the instrument’s built-in software version IMAGEnet 6 1.36. For the macular retinal and choroidal thicknesses, seven areas were analyzed: central (c), superior (s), inferior (i), superonasal (sn), inferonasal (in), superotemporal (st), and inferotemporal (it); the peripapillary choroidal thickness (ppCT) was quantified in four areas: superior, inferior, temporal, and nasal. The automatic segmentation algorithms of the OCT-angiography device were used. The choriocapillaris default segmentation was from Bruch’s membrane down to 20.8 μm. For the macular vascular density (VD), six areas (overall/total, fovea, superior, inferior, nasal, and temporal) were analyzed. The overall macular vascular density of the retina (rVDtot) and choriocapillaris (ccVDtot) was recorded as the mean of the foveal, superior, inferior, nasal, and temporal vascular density in each segment. The qualitative evaluation was performed by two investigators.

In the CEA group, the same procedure using bovine patch angioplasty was performed on all patients. CAS was performed through a femoral approach using filtering cerebral protection devices.

The quantitative variables are expressed as mean values (SD), while qualitative variables are expressed as absolute and relative frequencies. Paired *t*-tests or Wilcoxon signed-rank tests were used for time comparisons for the whole sample. For the comparison of proportions, the chi-square and Fisher’s exact tests were used. Student’s *t*-tests were computed for comparisons between the two groups. Time comparisons were analyzed using the Wilcoxon signed-rank test. Repeated measures analysis of variance (ANOVA) was adopted to evaluate the changes in the indexes of the two types of revascularization over the follow-up period. Log transformations were used in the repeated measures analysis of variance. All reported *p*-values are two-tailed. Statistical significance was set at *p* < 0.05, and the analyses were conducted using the SPSS statistical software (version 26.0).

## 3. Results

We initially examined 81 patients. Twenty-one of them were excluded because of an underlying, previously undiagnosed ocular disease, and eight of them were excluded because of their inability to cooperate in order to acquire sufficient quality OCT exams. Fifty-two patients (82.7% males) with a mean age of 68.4 years (SD = 7.9 years) were analyzed. There were 35 patients (67.3%) in the CEA group and 17 patients (32.7%) in the CAS group. Table 1 provides the participant demographic information as well as information regarding their medical histories. The patient characteristics of the two groups were similar.

The postoperative changes in the ipsilateral eyes are presented in Table 2. The ppCTi, MTs, SFCT, rVDn, rVDtot, ccVDt, ccVDn, and ccVDtot increased significantly after revascularization, while MTst and MTit decreased significantly after revascularization (CEA and CAS).

Table 3 presents the postoperative changes in the contralateral eyes. The MTsn, subfoveal choroidal thickness (SFCT), superior retinal vascular density (rVDs), rVDi, rVDt, rVDtot, ccVDs, ccVDt, and ccVDn increased significantly after revascularization (CEA and CAS).

The changes in the nerve fiber layer thickness, choroidal thickness around the optic nerve, and topographic measurements of the macula of the ipsilateral eye after each type of revascularization are presented in Table 4. The preoperative and postoperative values in the two groups were similar (*p* > 0.05). Regarding changes over time, it was found that in the CEA group, the ppCTs, ppCTi, and ppCTn increased significantly while MTst and MTit decreased significantly after revascularization.

**Figure 1:**
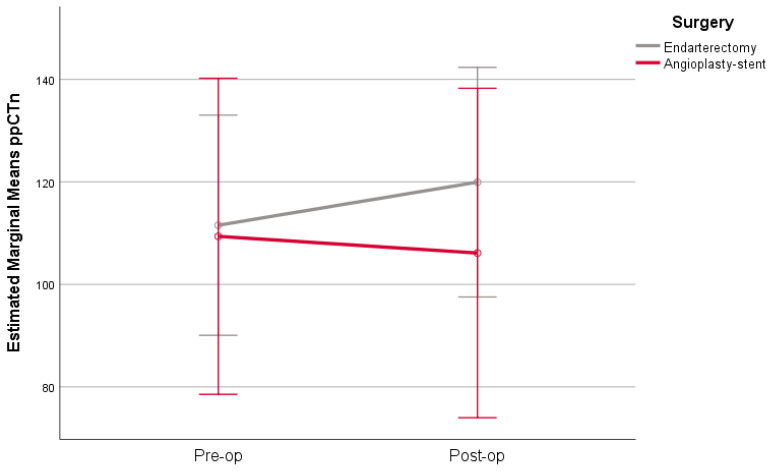
Changes in nasal peripapillary choroidal thickness (ppCTn) in ipsilateral eyes in the two revascularization groups; data are reported as the mean +/− SD.

In the CAS group, the central (foveal) MT (CMT) decreased significantly after revascularization. The degree of change in the ppCTn and MTst differed significantly between the two revascularization groups (*p* = 0.038 and *p* = 0.047, respectively). In the CEA group, the ppCTn values increased significantly, while they remained similar in the CAS group (Figure 1).

**Figure 2:**
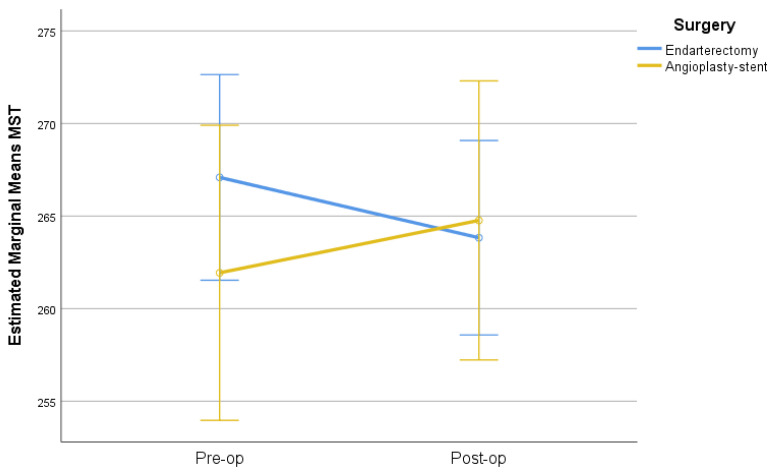
Changes in superotemporal macular thickness (MTst) in ipsilateral eyes in the two revascularization groups; data are reported as the mean +/− SD.

The MTst values also decreased significantly in the CEA group but did not change in the CAS group (Figure 2).

The changes in the macular choroidal thickness, retinal vascular density, and vascular density of the choriocapillaris of the ipsilateral eyes in the two revascularization groups are presented in Table 5.

**Figure 3:**
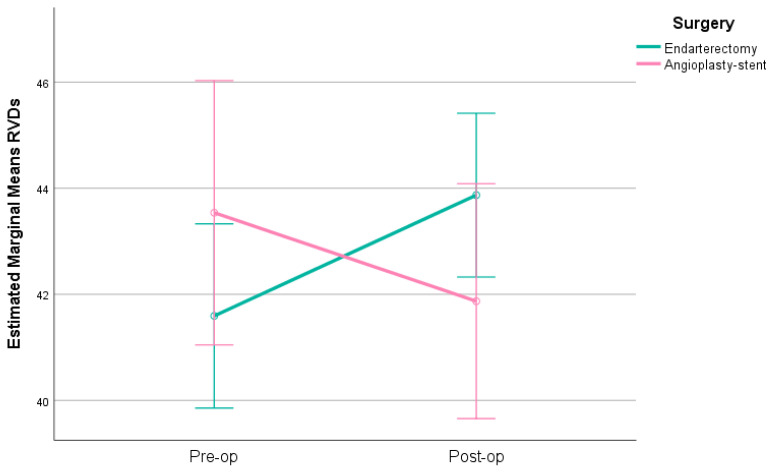
Changes in superior retinal vascular density (rVDs) in ipsilateral eyes in the two revascularization groups; data are reported as the mean +/− SD.

Interestingly, the SFCT, inferior choroidal thickness (CTi), inferonasal choroidal thickness (CTin), rVDs, rVDtot, and ccVDtot increased significantly after revascularization but only in the CEA group. Furthermore, ccVDt increased significantly after revascularization in both groups to a similar degree. Only the degree of change in the rVDs differed significantly between the two revascularization procedures (*p* = 0.007): it increased significantly in the CEA group and did not change in the CAS group (Figure 3).

## 4. Discussion

In the present study, we found a postoperative increase in choroidal and retinal thicknesses and vascular densities in the ipsilateral eye as a result of carotid artery revascularization. We also found an improvement in ocular perfusion, more specifically, a postoperative increase in SFCT and vascular density in the retina and choriocapillaris, in the contralateral eye. This is not surprising considering the anatomy of collateral orbital vessels. The development of the ophthalmic artery involves multiple anastomoses with both the internal and external carotid arteries. Multiple anastomoses are evident in the maxillary artery branch, an external carotid artery branch, and the distal branch of the ophthalmic artery [11,12], which often create a retrograde blood flow stemming from the external carotid artery in advanced ICA stenosis and ICA occlusion. The results of previous studies also support the hypothesis that revascularization to treat unilateral carotid stenosis can increase hemispheric cerebral blood flow on both sides [13]. It is also important to note that in our study, no significant inflammation was detected in either the CEA or CAS groups. Inflammation is a common concern following CAS and CEA, both aimed at treating carotid artery disease, but with distinct profiles of post-procedural responses. After CAS, inflammation often results from the physical disruption of plaques, injury to the artery lining, and the body’s immune response to the stent as a foreign object. This inflammatory process can lead to complications such as periprocedural ischemic lesions, restenosis, or in-stent restenosis, which involves the proliferation of cells narrowing the artery. Late inflammatory reactions may also occur because of prosthetic materials used during surgery. In our study, no significant inflammation was detected in either the CEA or CAS groups. In our patients, the typical inflammation at the incision site subsided within a few days without the use of antibiotics or steroids. Follow-up carotid artery ultrasound exams conducted one month after revascularization for both groups revealed a healthy, unobstructed carotid artery with no abnormalities.

Our study adds to the existing body of literature studying OCTA changes in patients with carotid artery stenosis undergoing treatment. Lahme et al. reported significantly improved flow density in the optic nerve head after carotid endarterectomy [14]. In their study, Cao et al. found that carotid artery stenting led to a significant increase in macular VD and choroidal vascular volume in a cohort of both asymptomatic and symptomatic patients (Cao et al.). Lee et al. were able to detect an increase in macular vessel densities in both eyes following unilateral CAS for severe carotid stenosis (Lee et al., Sci Rep). Our study is, in fact, the first to investigate the vascular changes in the retina and choriocapillaris after carotid revascularization in a cohort of asymptomatic patients with severe carotid artery stenosis undergoing CEA and CAS.

Comparing the two study groups, those undergoing CEA and those in the CAS group. There was a statistically significant ipsilateral increase in the ppCTs, ppCTi, ppCTn, SFCT, CTi, CTin, rVDs, rVDtot, and ccVDtot in the CEA group, suggesting improved ocular perfusion and indicating that CEA is inferior in terms of improving ocular perfusion in asymptomatic severe carotid artery disease. Interestingly, until now, this increase in postoperative choroidal thickness was only noted in cases where the extent of the stenosis was 50–70% but not in patients with a higher degree of stenosis [15]. In our study, we found a statistically significant postoperative increase in the peripapillary and macular choroidal thickness in patients with severe (70–80%) asymptomatic carotid disease.

Remarkably, the superotemporal and inferotemporal retinal macular thickness (MTst and MTit) decreased significantly after surgery in the CEA group. A possible explanation for this segmental decrease in macular retinal thickness could be the regression of preoperative functional hyperemia because of retinal vascular autoregulation in order to maintain an adequate oxygen supply. Interestingly, the retinal microcirculation has end arteries without anastomoses, which play barrier and autoregulatory roles, and is a relatively low-flow/high-oxygen extraction system [16]. The retina has small local oxygen and energy reserves and therefore, in conditions of hypoperfusion, functional hyperemia could counter the metabolic demands. This theory is also supported by the findings of Akcay et al., who investigated alterations in the choroid in patients with severe carotid disease and found a compensatory SFCT increase in ipsilateral internal carotid stenoses greater than 70% [17].

Furthermore, the significant rise in the aforementioned parameters suggests that carotid revascularization improves the ocular microcirculation, reduces the risk of developing an ocular ischemic syndrome, and indicates a positive prognosis for the patients’ visual function.

Previous studies explored the changes in CT as well as retinal and choroidal vascular changes, using the choroidal vascular index, before and after carotid artery revascularization (CEA or CAS). Initially, those studies measured the flow in the ophthalmic artery and later the macular and choroidal thickness. In 2015, Sayin et al. were the first to show that the macular and choroidal thicknesses were reduced in the eyes ipsilateral to the carotid stenosis, and later, a weak negative correlation between the mean macular thickness and the duration and percentage of the ICA stenosis was found [18,19]. CEA led to a significant increase in the SFCT, which was more noticeable in the ipsilateral eye [20]. It was shown that CAS also has the potential to improve the SFCT [21] as well as the vascular density of the deep vascular retinal complex in both eyes [22]. Severe and long-lasting ICA stenosis can potentially alter choroidal vessels so that no change in SFCT will occur [23]. Reversed blood flow in the ophthalmic artery, which can be observed in ophthalmic artery Doppler waveforms, is a predictor for advanced ICA stenosis with poor functional outcomes [24]. Although retrograde blood flow in the ophthalmic artery can fully recover after carotid revascularization [25], ocular function remains poor. This observation is supported by other studies that showed that chronic high-grade carotid artery stenosis in combination with retrograde ophthalmic artery blood flow leads to a significant and chronic reduction in blood flow in the ipsilateral central retinal artery [26,27]. The clinical consequence of the chronic reduction in blood flow is hypoperfusion retinopathy, progressing to ocular ischemic syndrome with devastating ocular results, such as ocular ischemia, retinal neovascularization, and neovascular glaucoma with secondary angle closure. OCTA and the evaluation of ocular microvasculature could be essential in the assessment of therapy success and could be used as noninvasive indicators to monitor asymptomatic carotid artery stenosis [28,29].

Although OCTA segmentation algorithms worked well, we had to manually correct the sclerochoroidal segmentation in three patients. This was counterbalanced by the fact that two graders showed complete agreement, and there was high intraindividual repeatability. Furthermore, OCTA is prone to certain imaging artifacts [30]. Nevertheless, only high-quality images were used in the analysis.

A small sample size is more affected by random fluctuations and outliers and is prone to the risk of type I and type II errors. Therefore, the small number of participants in our study constitutes a limitation. Despite the short follow-up period, our findings are supported by recent studies that suggested that improvements in choroidal thickness and vascular flow can be seen in the early postsurgical period [31]. Another limitation of our study is that, despite only including eyes with a refractive status of ±3D and measuring the CT at the same time of day to avoid diurnal effects on the CT, specific ocular biometric parameters such as axial length, anterior chamber depth or lens thickness that could modify the CT [32,33] and the accuracy of the OCTA findings were not measured. Moreover, a further limitation is that the vasculature of the retina and choriocapillaris was only analyzed in two dimensions from the OCTA scans, whereas the vasculature exhibits a three-dimensional configuration. Larger studies could allow for not only intrasubject but also intersubject comparisons and the development of software capable of examining the volume of the retinal and choriocapillaris vasculature. A complementary evaluation of the flow direction in the ophthalmic artery would also provide a more accurate answer to the question of which surgical procedure is more promising in terms of improving ocular perfusion.

Since the optimal treatment of asymptomatic carotid artery disease remains under debate, the examination of the retinal and choroidal microcirculation could be utilized as an additional biomarker to evaluate the clinical consequences of each surgical procedure and inform decision-making regarding selecting a surgical procedure. Determining the differences in ocular vascular structures is therefore essential for predicting long-term visual prognosis, preventing complications such as ocular ischemic syndrome, and preserving ocular function and also patients’ survival, since the retinal and choroidal microvasculature can be considered a window into the cerebral microvascular systems. Studying retinal microvasculature provides a unique opportunity for studying cerebral small vessel disease since cerebral microcirculation and retinal microvessels share similar anatomy, physiology, and embryology [34]. The direct visualization of retinal vessels to study retinal microvascular health as a proxy for brain microvascular health in patients with carotid artery disease could have many benefits [35]. Therefore, the retinal and choroidal microcirculation could serve as biomarkers for the severity of underlying cardiovascular, neurodegenerative, and microvascular diseases [36].

## Figures and Tables

**Table 1:** Cohort characteristics.

		Total Sample (*N* = 52; 100%)	Revascularization		*p*
			CEA (*N* = 35; 67.3%)	CAS (*N* = 17; 32.7%)	
		Ν (%)	Ν (%)	Ν (%)	
Gender					
	Male	43 (82.7)	28 (80.0)	15 (88.2)	0.700 ++
	Female	9 (17.3)	7 (20.0)	2 (11.8)	
Age, mean (SD)		68.4 (7.9)	67.9 (8.2)	69.3 (7.3)	0.560 ‡
Coronary heart disease		22 (42.3)	13 (37.1)	9 (52.9)	0.279 +
Hypertension		47 (90.4)	30 (85.7)	17 (100)	0.159 ++
Hypercholesterolemia		52 (100)	35 (100)	17 (100)	-
Diabetes		17 (32.7)	13 (37.1)	4 (23.5)	0.326 +
Peripheral artery disease		3 (5.9)	2 (5.9)	1 (5.9)	>0.999 ++
Nicotine use		29 (56.9)	20 (58.8)	9 (52.9)	0.689 +
Bilateral carotid stenosis		26 (50)	17 (48.6)	9 (52.9)	0.768 +
Side					
	Left	22 (42.3)	12 (34.3)	10 (58.8)	0.093 +
	Right	30 (57.7)	23 (65.7)	7 (41.2)	

+ Pearson’s chi-square test; ++ Fisher’s exact test; ‡ Student’s *t*-test.

**Table 2:** Changes in eyes ipsilateral to carotid artery revascularization, showing significant values in bold.

	Pre-Revascularization		Post-Revascularization		Change	*p* (Wilcoxon Signed-Rank Test)
	Mean (SD)	Median (IQR)	Mean (SD)	Median (IQR)	Mean (SD)	
ppCTs	119.7 (70.9)	89.5 (67–160)	123.3 (69.3)	97 (75.5–154)	3.6 (15.2)	0.100
ppCTi	98.6 (62.5)	79.5 (57–128.5)	104.6 (62.3)	86 (65.5–131)	6 (16.6)	**0.004**
ppCTt	117.1 (64.7)	103 (62.5–163.5)	117 (63.7)	102 (67.5–159.5)	−0.1 (27)	0.694
ppCTn	110.8 (62.7)	89 (62–129)	115.4 (65.7)	95 (65.5–132)	4.6 (21.6)	0.117
CMT	247.8 (23.7)	248.1 (232.5–259)	246.9 (24.5)	247.5 (232.5–258)	−1 (16.3)	0.070
MTs	276.8 (18.4)	277.3 (267.5–289)	278.1 (24.3)	276.5 (265–287)	1.3 (18)	**0.049**
MTi	274.4 (17.4)	274.5 (263.5–284)	271.4 (16.1)	271 (260–283)	−3 (9.5)	0.066
MTst	265.4 (16.4)	267.5 (257.5–275.5)	264.1 (15.3)	265.5 (255.5–274)	−1.3 (10.6)	**0.021**
MTit	268.1 (14.8)	269 (258–279.5)	265.9 (14.2)	265.5 (255.5–277)	−2.2 (7.4)	**0.013**
MTsn	288.3 (21.3)	287.5 (276–300.5)	286.6 (18.1)	285.5 (275–298.5)	−1.7 (11.1)	0.068
MTin	286.2 (17.8)	286 (276.5–296.5)	286 (19.4)	285.5 (275.5–296)	−0.2 (7.3)	0.783
SFCT	212 (93.7)	201.4 (131–281.5)	220.9 (92.2)	204 (138.5–292.5)	8.9 (30.2)	**0.011**
CTs	225.8 (68.5)	230.7 (190.5–240.5)	220.4 (66.7)	228.6 (176–235.5)	−5.4 (27.2)	0.383
CTi	227.8 (89.6)	234.6 (178.5–254)	226.7 (93.1)	237.2 (164–256)	−1.1 (24.3)	0.201
CTst	216.5 (60.3)	215.2 (200.5–239.5)	209.8 (60.5)	210.8 (176–230)	−6.7 (31.6)	0.062
Ctit	219 (75.6)	221.5 (181.5–268.5)	219.9 (81)	221.6 (160.5–283.5)	0.9 (30.3)	0.474
CTsn	187.3 (78.5)	193 (146.5–198.1)	187 (77.2)	197.5 (135–204.5)	−0.3 (24.7)	0.498
CTin	186.6 (84.1)	191.4 (139–196.2)	186.2 (84.6)	192.6 (130–205)	−0.4 (19.2)	0.206
rVDc	18.3 (5)	18 (15.4–21.5)	18.4 (5.1)	17.6 (15.1–21.2)	0.1 (4.6)	0.848
rVDs	42.2 (5.1)	42.1 (41–44.1)	43.2 (4.6)	43.6 (41.5–44.7)	1 (4.6)	0.140
rVDi	42 (4.7)	42.3 (40.8–44.6)	42.5 (4.4)	42.7 (40.6–44.4)	0.5 (4.6)	0.358
rVDt	42 (3.7)	42 (40.9–43.5)	42.6 (3.5)	42.7 (40–43.3)	0.6 (3.6)	0.343
rVDn	40.6 (5.9)	40.4 (39–41.8)	41.7 (6)	41.7 (39.7–43.4)	1.2 (5.2)	**0.033**
rVDtot	37 (3.6)	36.8 (35.8–38.3)	37.7 (3.3)	37.8 (35.8–39.2)	0.7 (2.7)	**0.018**
ccVDc	50.2 (6.8)	49.6 (46.3–52.5)	49.8 (5.7)	49.8 (48.2–52.7)	−0.4 (5.5)	0.891
ccVDs	49.3 (4.9)	49.2 (48.7–51)	50.1 (3.4)	50.2 (49.2–51.3)	0.8 (3)	0.092
ccVDi	49.8 (5.3)	50.8 (49.2–52.2)	50.2 (3.2)	50.3 (48.6–51.8)	0.3 (4.4)	0.967
ccVDt	51.1 (3.8)	51.3 (50.4–52.5)	52.2 (2.9)	52.2 (51–53)	1.1 (2.7)	**0.001**
ccVDn	51 (5.1)	51.2 (50.7–53.1)	52.1 (4)	52.4 (50.9–53.6)	1.1 (3.6)	**0.049**
ccVDtot	50.3 (3)	50.4 (49.7–51.6)	50.9 (2.1)	50.7 (50.4–51.8)	0.6 (1.7)	**0.015**

SD: standard deviation; ppCTs: superior peripapillary choroidal thickness; ppCTi: inferior peripapillary choroidal thickness; ppCTt: temporal peripapillary choroidal thickness; ppCTn: nasal peripapillary choroidal thickness; CMT: central macular thickness; MTs: superior macular thickness; MTi: inferior macular thickness; MTst: superotemporal macular thickness; MTit: inferotemporal macular thickness; MTsn: superonasal macular thickness; MTin: inferonasal macular thickness; SFCT: subfoveal choroidal thickness; CTs: superior choroidal thickness; CTi: inferior choroidal thickness; CTst: superotemporal choroidal thickness; CTit: choroidal thickness inferotemporal; CTsn: choroidal thickness superonasal; CTin: choroidal thickness inferonasal; rVDc: central retinal vascular density; rVDs: superior retinal vascular density; rVDi: inferior retinal vascular density; rVDt: temporal retinal vascular density; rVDn: nasal retinal vascular density; rVDtot: total overall average retinal vascular density; ccVDc: central choriocapillaris vascular density; ccVDs: superior choriocapillaris vascular density; ccVDi: inferior choriocapillaris vascular density; ccVDt: temporal choriocapillaris vascular density; ccVDn: nasal choriocapillaris vascular density; ccVDtot: total overall average choriocapillaris vascular density.

**Table 3:** Changes in eyes contralateral to the carotid artery revascularization, showing significant values in bold.

	Pre-Revascularization		Post-Revascularization		Change	*p* (Wilcoxon Signed-Rank Test)
	Mean (SD)	Median (IQR)	Mean (SD)	Median (IQR)	Mean (SD)	
ppCTs	123.1 (59.5)	108 (80–152)	123.1 (59.5)	111 (75.5–155.5)	0 (15.4)	0.535
ppCTi	93.4 (58.9)	84.5 (54–106)	93.9 (58.5)	84 (54–105)	0.5 (14.3)	0.963
ppCTt	111.5 (58.2)	103.5 (70–132)	115.4 (64.5)	103.5 (70.5–127)	3.9 (22.6)	0.265
ppCTn	115.8 (56.1)	105.5 (70.5–146.5)	112.5 (57.1)	100.5 (69–138.5)	−3.3 (26.2)	0.622
CMT	246.6 (25.2)	249 (234.5–262.5)	245.8 (25.2)	247 (229.5–262.5)	−0.8 (13)	0.177
MTs	271.8 (24.8)	273.5 (267–284.5)	277 (17.7)	277.5 (268–286.5)	5.2 (19.6)	0.161
MTi	271 (18.2)	271.5 (260.5–280)	269.4 (18.5)	272 (258.5–279)	−1.6 (8)	0.136
MTst	260.5 (23.1)	263.5 (255.5–274.5)	265.4 (15.7)	265 (258.5–275.5)	4.9 (18.5)	0.152
MTit	266.3 (15.8)	270 (258.5–274.5)	266.3 (16.5)	269 (257.5–275)	0 (4.9)	0.895
MTsn	285.4 (21)	285 (276.5–296)	286.6 (20.8)	287.5 (276.5–297.2)	1.2 (8.2)	**0.042**
MTin	283.6 (21.9)	285.5 (272–294.5)	283.4 (21.2)	284 (272.5–292.8)	−0.2 (6.6)	0.975
SFCT	223.8 (85)	217 (160.5–265)	230.3 (83.7)	220 (166–272)	6.5 (20)	**0.050**
CTs	221.2 (62.9)	228.7 (197.5–237)	224.4 (65.8)	235.1 (193.5–238)	3.2 (16.7)	0.091
CTi	216.9 (80.1)	224.3 (181.5–249.5)	212.9 (78.8)	225.4 (169–248.5)	−4 (20)	0.359
CTst	211.4 (63)	218.3 (184.5–229.5)	215 (65)	221.4 (186.5–229.5)	3.6 (18.7)	0.076
CTit	215.6 (68)	219.1 (175.5–235.5)	213.6 (68.4)	219.8 (163.5–231)	−2 (15.9)	0.547
CTsn	187.8 (76.7)	202.7 (142.5–205.4)	186.8 (77.1)	200.1 (134–205)	−1.1 (22.1)	0.359
CTin	178.8 (70.7)	187.5 (139–195.2)	181.1 (80.8)	195.5 (124–196)	2.3 (25.9)	0.355
rVDc	18.2 (4.3)	17.4 (15.9–19.8)	18.5 (4.5)	17.7 (16.9–20.6)	0.3 (2.8)	0.268
rVDs	40.3 (4.9)	40.3 (39.5–42.6)	41.8 (3.5)	41.9 (41.1–44)	1.6 (4.5)	**0.022**
rVDi	40.7 (4.3)	40.7 (38.4–42.9)	42.3 (8.9)	42.2 (40.5–44)	1.6 (9.8)	**0.003**
rVDt	40.7 (3.6)	40.7 (39.5–43)	41.7 (3.4)	42.2 (40.2–43.8)	1 (3.8)	**0.043**
rVDn	40 (4.7)	40.3 (38.1–42.3)	40.6 (3.5)	41.1 (39.7–42.5)	0.7 (4.1)	0.133
rVDtot	36 (2.8)	36.2 (35.2–37.5)	37 (2.6)	37 (35.5–38.1)	1 (2.2)	**0.001**
ccVDc	49.3 (4.8)	49.4 (46.9–51.5)	49.9 (4.3)	49.5 (47.6–52.2)	0.6 (4.7)	0.530
ccVDs	48.7 (3.2)	48.9 (48.1–50.7)	49.7 (3.7)	50 (49.5–51.3)	1 (3.6)	**0.005**
ccVDi	49.7 (3.3)	50.3 (48.3–51.3)	49.7 (3.4)	50.2 (48.7–51.7)	0.1 (3.8)	0.330
ccVDt	51.4 (2.5)	51.6 (50.8–52.8)	52 (2.7)	52.2 (51.2–52.7)	0.6 (2.3)	**0.018**
ccVDn	51.1 (3.7)	51.4 (49–53.4)	51.8 (3.6)	51.8 (51–53.4)	0.7 (2.7)	**0.038**
ccVDtot	50 (2.3)	50.3 (49.4–51.3)	50.6 (2)	50.7 (49.8–51.5)	0.6 (2.1)	0.077

SD: standard deviation; ppCTs: superior peripapillary choroidal thickness; ppCTi: inferior peripapillary choroidal thickness; ppCTt: temporal peripapillary choroidal thickness; ppCTn: nasal peripapillary choroidal thickness; CMT: central macular thickness; MTs: superior macular thickness; MTi: inferior macular thickness; MTst: superotemporal macular thickness; MTit: inferotemporal macular thickness; MTsn: superonasal macular thickness; MTin: inferonasal macular thickness; SFCT: subfoveal choroidal thickness; CTs: superior choroidal thickness; CTi: inferior choroidal thickness; CTst: superotemporal choroidal thickness; CTit: choroidal thickness inferotemporal; CTsn: choroidal thickness superonasal; CTin: choroidal thickness inferonasal; rVDc: central retinal vascular density; rVDs: superior retinal vascular density; rVDi: inferior retinal vascular density; rVDt: temporal retinal vascular density; rVDn: nasal retinal vascular density; rVDtot: total overall average retinal vascular density; ccVDc: central choriocapillaris vascular density; ccVDs: superior choriocapillaris vascular density; ccVDi: inferior choriocapillaris vascular density; ccVDt: temporal choriocapillaris vascular density; ccVDn: nasal choriocapillaris vascular density; ccVDtot: total overall average choriocapillaris vascular density.

**Table 4:** Ipsilateral changes in nerve fiber layer thickness, peripapillary choroidal thickness, and topographic measurements of the macula after CEA and CAS, showing significant values in bold.

Revascularization		Pre-Revascularization		Post-Revascularization		Change	*p* ^1^	*p* ^2^
		Mean (SD)	Median (IQR)	Mean (SD)	Median (IQR)	Mean (SD)		
RNFLtot	CEA	104.9 (15.6)	104 (97–116)	104.3 (14.6)	104 (93–115)	−0.6 (3.9)	0.647	0.356
	CAS	102.9 (8.9)	103 (102–107)	103.5 (9.6)	103 (101–109)	0.5 (3.6)	0.698	
RNFLs	CEA	130 (41.4)	130 (106–144)	124.6 (23.5)	125 (106–142)	−5.4 (29.9)	0.575	0.511
	CAS	124.7 (17.6)	124 (112–140)	124.2 (17.9)	125 (110–134)	−0.5 (6.3)	0.637	
RNFLi	CEA	140.4 (41.3)	139 (121–149)	135 (20.2)	140 (121–152)	−5.4 (35.3)	0.967	0.260
	CAS	129.1 (14.1)	130 (128–135)	132.2 (15)	131 (128–135)	3.1 (8.4)	0.452	
RNFLt	CEA	79.8 (42.7)	72 (65–83)	74.7 (17.3)	72 (65–81)	−5.1 (29.3)	0.212	0.497
	CAS	71.3 (8.6)	71.3 (64–79)	71.5 (9.6)	72 (63–81)	0.2 (3.6)	0.469	
RNFLn	CEA	90.3 (40)	85 (76–93)	84 (15.1)	83 (73–94)	−6.3 (31.3)	0.157	0.505
	CAS	87.4 (14.7)	86 (81–96)	86.5 (15.8)	87 (76–93)	−0.9 (4.5)	0.420	
ppCTs	CEA	119.9 (77.4)	87 (67–161)	125.1 (73.1)	93 (77–155)	5.2 (16)	**0.030**	0.090
	CAS	119.3 (57.1)	106 (67–160)	119.6 (62.8)	98 (70–153)	0.4 (13.1)	0.925	
ppCTi	CEA	100.7 (64.4)	79 (61–133)	109.4 (61.5)	96 (70–139)	8.8 (17)	**0.002**	0.127
	CAS	94.5 (60)	81 (44–118)	94.8 (64.6)	75 (48–118)	0.3 (14.6)	0.460	
ppCTt	CEA	115.3 (63.2)	101 (57–165)	116.9 (65.8)	102 (68–165)	1.6 (27.7)	0.674	0.526
	CAS	120.8 (69.4)	120.8 (69–148)	117.1 (61)	109 (63–154)	−3.7 (26)	0.924	
ppCTn	CEA	111.5 (63.9)	104 (61–125)	119.9 (65.2)	112 (70–134)	8.4 (22.3)	**0.034**	**0.038**
	CAS	109.4 (62)	85 (63–133)	106.1 (67.7)	79 (63–113)	−3.3 (18.3)	0.649	
CMT	CEA	247.6 (26.6)	248 (228–261)	247 (27.8)	248 (232–261)	−0.6 (19.9)	0.398	0.856
	CAS	248.3 (16.9)	248.3 (240–254)	246.5 (16.3)	247 (239–250)	−1.7 (3.1)	**0.037**	
MTs	CEA	276.5 (17)	277 (265–290)	278 (27.6)	276 (260–288)	1.4 (20.4)	0.111	0.892
	CAS	277.3 (21.5)	277.3 (270–286)	278.4 (16.2)	279 (266–281)	1 (12.5)	0.255	
MTi	CEA	275 (19.3)	276 (264–284)	272.5 (17)	273 (260–284)	−2.6 (9.8)	0.289	0.536
	CAS	273.2 (13)	273.2 (263–283)	269.2 (14.3)	266 (257–274)	−4 (8.9)	0.100	
MTst	CEA	267.1 (14.6)	268 (258–277)	263.8 (16.1)	266 (252–275)	−3.3 (8.7)	**0.008**	**0.047**
	CAS	261.9 (19.6)	263 (255–274)	264.8 (14.1)	264 (256–272)	2.8 (13.1)	0.756	
MTit	CEA	269.5 (15.1)	269 (259–280)	266.5 (15)	267 (254–278)	−3 (7.7)	**0.010**	0.280
	CAS	265.4 (14.1)	267 (256–272)	264.8 (12.7)	265 (256–271)	−0.6 (6.5)	0.488	
MTsn	CEA	287.2 (22.6)	287 (275–302)	285 (18.6)	286 (272–300)	−2.2 (11.4)	0.122	0.655
	CAS	290.6 (18.8)	290.6 (282–300)	289.9 (17.1)	285 (279–297)	−0.7 (10.7)	0.343	
MTin	CEA	284.5 (18.2)	284 (276–295)	285.6 (20.1)	289 (275–296)	1.1 (6.4)	0.329	0.075
	CAS	289.7 (16.9)	289.7 (277–298)	286.9 (18.2)	283 (276–296)	−2.8 (8.4)	0.344	

^1^ *p*-value for time effect (Wilcoxon signed-rank test). ^2^ Repeated measures ANOVA *p*-value (using logarithmic transformations) for the time × surgery effect. SD: standard deviation; RNFLtot: overall average thickness of retinal nerve fiber layer; RNFLs: thickness of superior retinal nerve fiber layer; RNFLi: thickness of inferior retinal nerve fiber layer; RNFLt: thickness of temporal retinal nerve fiber layer; RNFLn: thickness of nasal retinal nerve fiber layer; ppCTs: superior peripapillary choroidal thickness; ppCTi: inferior peripapillary choroidal thickness; ppCTt: temporal peripapillary choroidal thickness; ppCTn: nasal peripapillary choroidal thickness; CMT: central macular thickness; MTs: superior macular thickness; MTi: inferior macular thickness; MTst: superotemporal macular thickness; MTit: inferotemporal macular thickness; MTsn: superonasal macular thickness; MTin: inferonasal macular thickness.

**Table 5:** Changes in subfoveal and choroidal thickness, flow in retinal vessels, and choriocapillaris blood flow of the ipsilateral eye after CEA and CAS.

		Pre		Post		Change	*p* ^1^	*p* ^2^
		Mean (SD)	Median (IQR)	Mean (SD)	Median (IQR)	Mean (SD)		
SFCT	CEA	213.5 (95.8)	194 (130–284)	223.1 (95)	206 (134–300)	9.6 (28.7)	**0.011**	0.928
	CAS	208.9 (92.1)	208.9 (134–254)	216.5 (88.8)	202 (163–266)	7.6 (34.1)	0.407	
CTs	CEA	230.7 (62.6)	230.7 (198–239)	228.5 (60.9)	228.6 (203–230)	−2.2 (19.3)	0.723	0.168
	CAS	215.5 (80.3)	215.5 (168–242)	203.6 (76.6)	203.6 (147–245)	−11.9 (38.7)	0.246	
Cti	CEA	234.6 (82.2)	234.6 (206–242)	237.2 (82.6)	237.2 (186–249)	2.6 (17.2)	**0.001**	0.075
	CAS	213.7 (104.6)	213.7 (143–262)	205.1 (111.4)	191 (135–261)	−8.6 (34.1)	0.276	
CTst	CEA	215.2 (51.6)	215.2 (203–228)	210.8 (52.5)	210.8 (189–223)	−4.5 (20.1)	0.171	0.470
	CAS	219.2 (77)	219.2 (165–263)	207.8 (76.2)	207.8 (163–231)	−11.4 (47.9)	0.237	
CTit	CEA	221.5 (67.9)	221.5 (190–260)	221.6 (64)	221.6 (176–281)	0.1 (17.6)	0.056	0.529
	CAS	213.8 (91.6)	213.8 (165–299)	216.3 (110.3)	204 (144–298)	2.5 (47.6)	0.538	
CTsn	CEA	198.1 (74.7)	198.1 (157–214)	198.1 (76.1)	198.1 (153–211)	0 (17.2)	0.617	0.963
	CAS	165 (83.7)	155 (123–165)	164.1 (76.6)	151 (128–197)	−0.9 (36.3)	0.776	
CTin	CEA	191.4 (80)	191.4 (143–201)	192.5 (80.1)	192.6 (142–206)	1.1 (15.2)	**0.045**	0.383
	CAS	176.5 (93.7)	176.5 (125–182)	173 (94.3)	170 (116–204)	−3.5 (25.9)	0.943	
rVDc	CEA	18 (4.9)	18 (15.3–20.8)	17.6 (4.7)	17.6 (14.5–21.2)	−0.4 (4.9)	0.646	0.321
	CAS	19 (5.2)	19 (15.4–22.1)	20 (5.5)	20 (16.6–22.5)	1 (3.9)	0.554	
rVDs	CEA	41.6 (5.9)	41.6 (39.3–44.3)	43.9 (4.8)	43.9 (41.2–45.9)	2.3 (4.6)	**0.004**	**0.007**
	CAS	43.5 (2.8)	43.5 (42.3–43.9)	41.9 (3.9)	43.2 (41.9–44.4)	−1.7 (3.5)	0.102	
rVDi	CEA	41.4 (5.5)	41.4 (38.1–44.5)	42.3 (4.9)	42.3 (40.8–44.2)	0.9 (4.8)	0.094	0.326
	CAS	43.4 (1.9)	43.2 (41.8–44.7)	43 (3.3)	43 (40.5–44.9)	−0.4 (4.1)	0.434	
rVDt	CEA	42 (4)	42 (40.8–43.6)	43 (3.5)	43 (40–44.3)	1 (3.4)	0.159	0.254
	CAS	41.9 (3.2)	41.9 (41.3–43.2)	41.8 (3.5)	41.8 (39.7–42.9)	−0.1 (3.9)	0.653	
rVDn	CEA	41.2 (6.5)	41.2 (38.9–42.7)	42.1 (6.5)	42.1 (39.8–43.5)	0.9 (5.1)	0.225	0.590
	CAS	39.3 (4.5)	39.3 (39–40.5)	40.9 (4.7)	40.9 (38.7–41.8)	1.6 (5.4)	**0.049**	
rVDtot	CEA	36.8 (4.1)	36.8 (35.5–38.3)	37.8 (3.7)	37.8 (35.7–39.3)	0.9 (2.7)	**0.018**	0.261
	CAS	37.4 (2.2)	37.4 (36.8–38.3)	37.5 (2.6)	37.7 (37–38.5)	0.1 (2.6)	0.356	
ccVDc	CEA	49.2 (6.9)	49.2 (45.8–52.2)	48.8 (5.7)	49.5 (47.1–52.7)	−0.4 (6.1)	0.909	0.999
	CAS	52.3 (6.4)	51.6 (49.3–53.3)	51.8 (5.1)	51.6 (48.3–52.8)	−0.5 (4.3)	0.687	
ccVDs	CEA	49.2 (5.8)	49.2 (48.7–51.1)	50.2 (3.8)	50.2 (49.2–51.4)	1 (3.3)	0.088	0.341
	CAS	49.6 (2.1)	49.5 (48.7–50.2)	49.8 (2.3)	49.8 (49.3–50.1)	0.2 (2.4)	0.507	
ccVDi	CEA	49.4 (6.3)	49.4 (49.1–52.3)	49.7 (3.3)	49.7 (48.4–51.8)	0.3 (5.2)	0.961	0.853
	CAS	50.8 (1.6)	51 (50.1–51.7)	51.2 (2.7)	51.2 (49.4–52.3)	0.5 (2.5)	0.868	
ccVDt	CEA	51.3 (4.5)	51.3 (50.7–53.2)	52.4 (3.2)	52.3 (51.1–53)	1.1 (3.1)	**0.032**	0.797
	CAS	50.8 (1.7)	50.8 (49.7–51.5)	51.8 (2.3)	51.8 (50.7–53.1)	1.1 (1.7)	**0.015**	
ccVDn	CEA	51.1 (6)	51.4 (51–54.3)	52.4 (4.4)	52.4 (51.3–53.7)	1.3 (4)	0.127	0.562
	CAS	50.8 (2.4)	50.8 (49.9–52.6)	51.6 (3)	51.6 (50.5–53.4)	0.8 (2.9)	0.209	
ccVDtot	CEA	50 (3.4)	50.2 (49.8–51.6)	50.7 (2.4)	50.7 (50.3–51.5)	0.7 (1.8)	**0.011**	0.553
	CAS	50.9 (1.5)	50.9 (49.7–51.9)	51.3 (1.4)	51.3 (50.6–52.2)	0.4 (1.5)	0.381	

^1^ *p*-value for time effect (Wilcoxon signed-rank test). ^2^ Repeated measures ANOVA *p*-value (using logarithmic transformations) for the time × surgery effect. SFCT: subfoveal choroidal thickness; CTs: superior choroidal thickness; CTi: inferior choroidal thickness; CTst: superotemporal choroidal thickness; CTit: inferotemporal choroidal thickness; CTsn: superonasal choroidal thickness; CTin: inferonasal choroidal thickness; rVDc: central retinal vascular density; rVDs: superior retinal vascular density; rVDi: inferior retinal vascular density; rVDt: temporal retinal vascular density; rVDn: nasal retinal vascular density; rVDtot: total overall average retinal vascular density; ccVDc: central choriocapillaris vascular density; ccVDs: superior choriocapillaris vascular density; ccVDi: inferior choriocapillaris vascular density; ccVDt: temporal choriocapillaris vascular density; ccVDn: nasal choriocapillaris vascular density; ccVDtot: total overall average choriocapillaris vascular density.

## Data Availability

Data are available upon reasonable request.
